# Illustrations of the Botany, and Other Branches of Natural History, of the Himalayan Mountains, and of the Flora of Cashmere

**Published:** 1835-07-01

**Authors:** 


*Illustrations of the Botany, and other Branches of Natural History, of the Himalayan Mountains, and of the Flora of Cashmere.* By J. Forbes Royle, Esq., F.L.S., &c. Parts V. and VI.—London, 1835. Folio.

This truly splendid and admirable work acquires strength in its progress, like the fame that it will ensure its author. Nos. V. and VI. are worthy successors of No. IV., of which we gave a notice in our Review for January last. The first three Numbers we have not seen, but doubt not, judging from those before us, that they are meet partakers of our approbation. Ὡς αιει τον ὁμοιον αγει Θεος ὡς τον ὁμοιον.

These two Numbers contain figures of thirty-eight species of plants, of which many are exceedingly beautiful: moreover, it is to us one of the pleasing features of this work, that it treats of nature as nature is found, and does not, as is so common, destroy the interest of her works by isolation. Botanists are often blind to every thing but plants; geologists frequently disregard the surface, and can only see "five fathom deep;" and entomologists as commonly neglect both soil and vegetation, and concentrate their observations on a fly. Mr. Royle's work, it is true, is chiefly botanical, but the references made to other sciences, and the contributions levied in collateral districts are both numerous and valuable.

Of the plants now figured, several species of Saxifrage, especially S. ciliata, have attracted our attention. Parnassia nubicola is also very pretty, and Dolomiæa macrocephala and Eremostachys superba handsome. Indeed, we might enumerate the whole, but must content ourselves with stating our belief that this work will form a very acceptable and important addition to our previously acquired stores.

We subjoin an extract, as a specimen of the interesting matter which is interwoven with the more dry technical details.

"The grape-vine being a plant of so much value and importance, its distribution is an interesting subject of inquiry, though there is little prospect of its becoming in India of greater value than as affording an agreeable fruit; though this is of sufficient importance to render highly desirable the introduction and trial of different and superior kinds from Europe. The native country of the vine seems now to be better ascertained than that of many other as extensively cultivated plants. Bieberstein, in his *Flora Tauro-Caucasica* (1, p. 174,) states, 'Nusquam non præter alpestria, per omnem de qua sermonem facimus regionem sponte in sylvis atque dumetis nascitur, et altissimas quandoque arbores ascendens, totas quantas occupat.' The author of the 'Mukhzunool-udwieh,' who was an inhabitant of the district, describes the vine, as found both wild and in gardens at Tinkaboon, in Deilim, about lat. 37°, on the southern shores of the Caspian, and that it is there called *dewaz.* Humboldt, also, in his '*Geographie des Plantes,*' p. 26, mentions that the vine 'grows wild on the coasts of the Caspian Sea, in Armenia, and in Caramania. The species of *Vitis*, which are found wild in North America, and which gave the name of *Winenland* to the first part of the New Continent which Europeans discovered, are very different from our *Vitis vinifera.*' These, as we learn from Pursh, are *Vitis labrusca*, called fox-grape; *V. æstivalis*, summer-grape; and *V. cordifolia*, winter-grape. From the sacred writings we know that the grape was cultivated in Asia in the earliest periods. M. Bové, the latest scientific traveller, informs us (Ann. des Sc. Nat. 1834, p. 172,) that it is still cultivated, and a good wine made in the vicinity of Jerusalem; but that in Egypt he found wine made only at *Medinet-el-Fayoum* (l. c. p. 76) which is in lat. 22° 20′. 'From Asia,' Humboldt continues, 'it passed into Greece, and thence into Sicily. The Phocæans carried it into the south of France, the Romans planted it on the banks of the Rhine;' and we have it now extending to 51°, or even 52°, in England, where it ripens well, as in the present fine season, in the open air; and wine is made in a few places in Devonshire. Southward the vine extends as far as 12° of northern latitude; as we learn from Dr. Ainslie (Ind. Mat. Med. 1, p. 156,) that 'the French are particularly successful in cultivating the grape at Pondicherry, notwithstanding the great heat of the Carnatic.' The illustrious Humboldt, in his *Proleg. de distrib. Geograph. Plant.* p. 159, where, from the examination of a multitude of facts, he has deduced the requisites for the successful cultivation of many plants, has observed, that 'the vine in Europe yields a generous and excellent wine between the latitudes of 36° and 48°, where the mean annual temperature is from 62° to 50°, or even 47°.5, provided that of winter is not below 38°, nor that of summer below 66° or 68°. These conditions are fulfilled on the sea-coast as high as lat. 47°, in the interior as high as lat. 50°, and in North America only as high as lat. 40°. The vine may therefore be cultivated for wine in a belt of from 12° to 15° of latitude in breadth on both sides of the Line; though to a much greater extent, if required, for its fruit only: but for both purposes, in a narrower space in the New than in the Old World. Further north than 48° of latitude, grapes do not generally secrete sufficient saccharine matter to undergo a proper vinous fermentation, and further south than 35° (or 32° in an insular situation like Madeira) though they are both sweet and high-flavoured, the temperature is so great that the juice passes rapidly into the acetous fermentation; and therefore the grapes of the most southern parts of Europe are more frequently dried as raisins than converted into wine. The climate of India is such as to exclude it from benefiting either by preserving the grape, or converting it into wine; though in the north-western provinces, the vines thrive well, and bear abundantly. They flower in February, and ripen the fruit (which is well, though perhaps not so delicately flavoured as in more temperate climates) about the middle of June, or about the time the vine is said to flower in Caucasus: at this time the mean temperature being about 90°, is evidently much too great to allow of a slow and gradual vinous fermentation; while the accession of the rainy season immediately afterwards produces so great a degree of moisture, as to render it impossible to dry the grapes as raisins, unless this could be effected in ovens, after being plunged in boiling water, as is done in some parts of Europe. It might, perhaps, be practicable even to make wine by growing the grapes at the foot of the mountains, where free from jungles, as in the country beyond the Jumna, and conveying them to a moderate temperature on the mountain side. A brewery has been established in a situation where the mean temperature in the houses hardly ever varied from 60° in the warm weather, and the distance was so inconsiderable, that it was thought preferable to bring the barley from the plains, rather than use that which was grown on the spot. The Deyra Doon would be a particularly favourable situation; but at present there is too much uncleared jungle, and the climate too moist, to ripen the grape properly in the short season, from the middle of March to the middle of June; the greatest pains were taken in their cultivation, but without success, by the Hon. Mr. Shore while resident there.

"But it is observed, that when the warmth of a low latitude is compensated for by elevation, or a barrier is opposed to the inundating influence of the rainy season, grapes are ripened as fruit, dried as raisins, and converted into wine. Thus, in Kunawur, between N. lat. 31° and 32°, or nearly that of Madeira, where elevation produces the same moderation of temperature, that is, in the latter, the consequence of its insular situation, we have luxuriant vineyards between 9,000 and 10,000 feet of elevation, with grapes of delicious flavour, which the moderation of temperature in September allows of being converted into wine, and the dryness (v. p. 34) to be preserved as raisins. Two degrees further north, or in the valley of Cashmere, at an elevation of 5,500 feet, we have grapes both excellent and plentiful, as we learn from both Mr. Foster and Mr. Moorcroft. The latter says, that 'many thousands of acres skirting the foot of the hills, are covered with apple and pear trees in full bearing, but without owners.' (Jour. of Geog. Society, 1, p. 241 and 253.) My plant collectors expressed their admiration, by describing the fruit trees as forming a perfect jungle in Cashmere. The moderation of temperature, with the existence of moisture, has been mentioned at p. 27, as accounting for the magnitude attained by may species of European genera. This will also explain the great size of the vines, which Mr. Moorcroft informs us, 'scales the summit of the poplar,' as well as for the want of a fine flavour, observed in the grapes brought to India, packed in layers of cotton. At Khoten, also, the vine is described by Mr. Moorcroft, as being very productive. The different kinds of raisins called *monukka*, *kismish*, and *bedana*, are brought chiefly from Istaulik. At Cabool, nearly in the same latitude, but more to the eastward than Cashmere, and elevated 6,000 feet, the grapes are described by Lieut. Burnes to be so plentiful, as to be given for three months to cattle. They are also abundant at Bokhara, and in both places are converted into wine, and dried as raisins. Astrakhan, in 46° of N. latitude, seems to be the most northern point in Asia where the grape thrives, and there the vineyards are described as being numerous. Every traveller mentions the grapes and wine of Persia. Dr. Ainslie says, it was from thence, as well as from the banks of the Rhine, that grape-plants were originally sent to the Cape of Good Hope, and that some of these from Persia now produce the red and white Constantia. This is generally considered the only good wine from that settlement. Dr. Ainslie thinks highly of the Madeira, made from the *groene druyf;* but Pontac is also a good and very sound wine. The Persians, it may be added, claim the discovery of wine, and call it *zuhr-i-khoosh*, or the delightful poison." (P. 146.)

